# *Lactobacillus*-fermented feed alters growth performance, fecal short-chain fatty acid contents, metagenomics, and metabolomics in growing pigs

**DOI:** 10.1128/msystems.00404-26

**Published:** 2026-07-20

**Authors:** Hui Liu, Meixia Chen, Dongyan Zhang

**Affiliations:** 1 Institute of Animal Science and Veterinary Medicine, Beijing Academy of Agriculture and Forestry Sciences 107624 , Beijing, China; Colorado State University, Fort Collins, Colorado, USA

**Keywords:** *Lactobacillus*-fermented feed, growing pigs, growth performance, short-chain fatty acids, fecal metabolome, metagenomics

## Abstract

This study sought to comprehensively evaluate the impact of *Lactobacillus*-fermented feed produced with *Latilactobacillus curvatus* SQ13 on the growth and fecal short-chain fatty acid (SCFA) contents, as well as metagenomic and metabolomic parameters, in growing pigs. One hundred crossbred pigs were randomized into two dietary treatment groups. Animals were given either a basal diet (CON) or a diet containing *Lactobacillus*-fermented feed (LP) over a 32-day period. The LP group exhibited a significantly reduced feed conversion ratio (FCR) and lower fecal valeric acid concentration compared with the CON group (*P* < 0.05). Microbial community analysis revealed that the relative abundance levels of *Candidatus_Eremiobacterota*, *Cyanobacteriota*, *Lacrimispora*, and *Candidatus_Onthomorpha* were markedly increased in the LP group (*P* < 0.05), whereas *Actinomycetota*, Pseudomonadota, *Solobacterium*, and *Mitsuokella* were significantly decreased (*P* < 0.05). KEGG pathway analyses indicated that arachidonic acid metabolism, phototransduction—fly, and Th17 cell differentiation were the three most significantly altered pathways. Correlation analysis further demonstrated that FCR was positively associated with *Solobacterium* abundance and downregulated metabolites, while showing negative correlations with *Alistipes* and upregulated metabolites. Collectively, these findings suggest that *Lactobacillus*-fermented feed improves growth efficiency and modulates valeric acid levels in growing pigs, likely through coordinated alterations in gut microbial composition and host metabolic processes. These results offer a potential foundation for the application of fermented feed in swine production systems.

**IMPORTANCE:**

Our study demonstrated that fermented feed produced by *Latilactobacillus curvatus* ZLA031 could improve growth efficiency, decrease valeric acid levels in growing pigs, likely through coordinated alterations in fecal microbiota composition, and host metabolic processes. Our work provided both experimental evidence and a theoretical framework supporting the application of *Lactobacillus*-fermented feed in swine production, while highlighting the need for further mechanistic research.

## INTRODUCTION

As a common component of the intestinal microbiota, *Lactobacillus* species are vital for host health through the production of lactic acid during carbohydrate fermentation, thereby lowering environmental pH. Due to these properties, it has been widely considered a viable alternative to antibiotics in animal production systems. In recent years, *Lactobacillus*-fermented feed has gained increasing attention in swine production because of its favorable fermentation characteristics and associated health benefits. Microbial fermentation represents a fundamental biochemical process in which microorganisms utilize feed substrates for growth and generate various metabolites. These include short-chain fatty acids (SCFAs), enzymes, extracellular polysaccharides, and other bioactive compounds that can reduce feed pH and enhance its nutritional value (1, 2). It has been found that supplementation with appropriately formulated *Lactobacillus*-fermented feed can improve gut microbial balance in pigs (3), strengthen their intestinal barrier function (4), and promote their overall growth performance (5).

*Latilactobacillus curvatus* (*L. curvatus*), formerly classified within the genus *Lactobacillus*, is recognized as a promising probiotic candidate. This species is commonly isolated from fermented foods and the gastrointestinal tract of both animals and humans, and is noted for its robust fermentation capacity and beneficial physiological effects (6–8). Earlier studies have shown that the *L. curvatus* SQ13 strain from the feces of healthy growing pigs has demonstrated desirable probiotic traits *in vitro*, including tolerance to acidic and bile conditions, antimicrobial activity against pathogenic bacteria, and efficient lactic acid production.

Our prior work has shown that *Lactobacillus*-fermented feed can enhance growth performance in pigs by modulating SCFA composition, gut microbiota, and metabolic profiles (9, 10). The specific effects of feed fermented with *L. curvatus* on growing pigs have not, however, been fully assessed. Here, the effects of *L. curvatus* SQ13-fermented feed on growth performance, fecal SCFA concentrations, and host-associated metagenomic and metabolomic profiles were investigated. Additionally, the relationships among these variables were analyzed to provide a more integrated understanding of how fermented feed influences gut microbial ecology and host metabolism.

## MATERIALS AND METHODS

### Preparation of *Lactobacillus*-fermented feed

The strain *L. curvatus* SQ13 was identified using standard biochemical, morphological, and physiological assays, along with 16S rRNA sequencing, by the China Center of Industrial Culture Collection (Beijing, China), and has been deposited in the China General Microbiological Culture Collection Center (CGMCC No. 31018). For propagation, the strain was cultured for 24 h in MRS medium at 37°C before centrifugation (5,000 × *g,* 10 min, 4°C). The resulting pellet was resuspended in 20% (wt/vol) reconstituted skim milk before lyophilization in an Epsilon 2-60 freeze dryer (Martin Christ GmbH, Germany). The viable cell count of the dried powder was determined by spreading 0.1 mL of 10^−7^ and 10^−8^ dilutions on MRS agar plates, followed by incubation at 37°C for 48 h. The dried powder had a cell density of 1.0 × 10^10^ CFU/g and was stored at 4°C in sealed containers. The preparation method for fermented feed was as follows. The dried powder was mixed with the basic feed at a mass ratio of 3:100. Water was then added to a water-to-feed ratio of 1:1, followed by thorough mixing and fermentation at 37°C for 24 h. Fermentation was carried out in sterile, airtight plastic containers, and the fermented feed was prepared in the same manner each day. The final formulation contained 1.73 × 10^9^ CFU/g viable bacteria and had a pH of 4.32.

### Pigs and experimental groupings

One hundred growing Landrace × Large White pigs with a mean starting body weight (BW) of 25.93 ± 0.46 kg were randomly allocated to two groups, with 5 replicate pens, with 10 pigs per pen, included in each group. The diet formulations met or exceeded the nutritional requirements specified in the Chinese standard GB/T 39235-2020 for growing pigs (Table 1). The control group (CON) received a basal diet without antibiotics or probiotics, while the experimental group (LP) was given the basal diet containing 0.3% *Lactobacillus*-fermented feed. The feeding trial lasted 32 days, including a 5-day adaptation period. Feed and water were not restricted. Body weights were recorded at the start (day 0) and on completion (day 32) of the trial, while feed intake per pen was measured weekly. These data were used to calculate growth performance parameters as follows: average daily gain (ADG) = total body weight gain per pig (g)/number of experimental days; average daily feed intake (ADFI) = total feed intake per pig (g)/number of experimental days; and Feed conversion ratio (FCR) = total feed intake (g)/total body weight gain (g).

**TABLE 1:** Composition of basal diet of growing pigs (as-fed basis)

Characteristic	Contents
Ingredients (%)	
Corn	68
Soybean meal	25
Wheat bran	3
Premix^*a*^	4
Total	100
Nutrient levels	
Digestible energy,^*b*^ MJ/kg	14.20
Crude protein,^*c*^ %	17.24
Lysine,^*c*^ %	0.91
Methionine,^*c*^ %	0.27
Calcium,^*c*^ %	0.88
Total phosphorus,^*c*^ %	0.40

^*a*^ Vitamin and mineral premix provided per kilogram of complete diet: vitamin A, 8,500 IU; Vitamin D3, 825 IU; vitamin E, 50 mg; vitamin K3, 5 mg; vitamin B1, 5 mg; vitamin B2, 5 mg; Vitamin B6, 2 mg; vitamin B12, 25 mg; nicotinic acid, 25 mg; pantothenic acid, 50 mg; biotin, 0.2 mg; Cu, 50 mg; Fe, 80 mg; Zn, 100 mg; Mn, 20 mg; I, 0.5 mg; and Se, 0.20 mg.

^*b*^ Calculated value.

^*c*^ Measured value.

### Sample collection

After the feeding trial, fecal samples were collected from 20 random pigs (10 per treatment group; 2 pigs per pen with average body weight). Each sample was subdivided into three equal portions which were frozen in microcentrifuge tubes in liquid nitrogen and subsequently kept at −80°C for analyses of fecal SCFAs, metagenomic sequencing, and metabolomic profiling.

### Analysis of fecal SCFA levels

Fecal SCFA levels were quantified using a gas chromatograph (7890B, Agilent Technologies, Santa Clara, CA, USA), as described with minor modifications (3). Specifically, approximately 0.5 g of feces was suspended in 10 mL of distilled water, thoroughly vortexed, and centrifuged (12,000 × *g*, 10 min). The resulting supernatant was filtered (0.22 μm) prior to analysis. The chromatography involved the use of a DB-FFAP capillary column (30 m × 250 μm × 0.25 µm; Agilent). One microliter of each sample was injected using a 10:1 split ratio, the temperatures of both the injector and detector were 250°C, and nitrogen with a flow rate of 1 mL/min served as the carrier gas.

### Microbial DNA extraction, metagenomic sequencing, and functional annotation

Total genomic DNA was isolated from the samples using a QIAamp Microbiome Kit (Qiagen, Hilden, Germany). DNA quality and concentration were evaluated on 1.5% agarose gel electrophoresis and spectrophotometrically (NanoDrop 2000, Thermo Fisher Scientific, Waltham, MA, USA), respectively. The extracted DNA was sheared to an an average ~400 bp fragment sizes using a Covaris M220 system (Gene Company Limited, Hong Kong, China) for paired-end library preparation. Metagenomic sequencing was subsequently undertaken on a NovaSeq 6000 platform (Illumina, San Diego, CA, USA) at Majorbio Bio-Pharm Technology Co., Ltd. (Shanghai, China).

The raw reads were filtered for quality using fastp (version 0.23.1). MEGAHIT (v.1.1.2) was used for clean read assembly, with retention of contigs ≥300 bp for downstream analyses. MetaGene was utilized for the prediction of open reading frames (ORFs), while CD-HIT (v.4.6.1) was applied for the removal of redundant sequences using the thresholds of 95% sequence identity and 90% coverage to generate a non-redundant gene catalog. To estimate gene abundance, high-quality reads were aligned to predicted genes using SOAPAligner (v.2.21). Functional and taxonomic annotations were assigned by aligning the non-redundant gene set against the NCBI NR and KEGG databases using DIAMOND.

### Extraction and analysis of metabolites from fecal samples

Metabolite extraction was performed from 50 mg of fecal material using a methanol-water solution (4:1, vol/vol) with 0.02 mg/mL L-2-chlorophenylalanine as an internal standard. After vortexing for 30 s, sonication in an ice-water bath for 10 min, and centrifugation (12,000 × *g*, 15 min, 4°C), sample supernatants were retained for subsequent LC-MS analysis. Metabolomic analyses were performed using a Thermo UHPLC-Q Exactive HF-X system with an ACQUITY HSS T3 column (100 Å, 1.8 μm, 100 mm × 2.1 mm; Waters, USA) at Majorbio Bio-Pharm Technology Co., Ltd. After processing of the raw data using Progenesis QI (v.4.0), enabling peak detection, compounds were identified based on alignment with the HMDB, METLIN, and Majorbio databases. Multivariate principal component analysis (PCA) and orthogonal partial least squares discriminant analysis (OPLS-DA) were applied to evaluate sample distribution and group separation. Differential metabolites were identified using variable importance in projection (VIP) scores ≥1 from OPLS-DA combined with *t*-tests (*P* < 0.05).

### Statistical analysis

Growth performance data and fecal SCFA concentrations were analyzed using independent-samples *t*-tests in SPSS 25.0. For metagenomic data, differences in microbial taxa and functional features between groups were assessed using the Wilcoxon rank-sum test and linear discriminant analysis effect size (LEfSe). Untargeted metabolomic data were analyzed using *t*-tests. Spearman correlation coefficients were determined to assess associations. The significance threshold was *P* < 0.05. All graphical outputs were created using GraphPad Prism 9.0.

## RESULTS

### *Lactobacillus*-fermented feed improved growth performance

As presented in Table 2, FCR values were markedly lower in pigs in the LP group relative to those in the CON group (*P* < 0.05). However, there were no substantial differences in initial BW, final BW, ADG, and ADFI between the groups (*P* > 0.05).

**TABLE 2:** Growth performance of growing pigs fed with *Lactobacillus*-fermented diets^*a*^

Characteristic	CON	LP	SEM	*P* value
Initial body weight, kg	25.98	25.87	0.46	0.913
Final body weight, kg	51.18	52.86	1.18	0.507
Average daily weight gain, kg/day	0.79	0.84	0.03	0.316
Average daily feed intake, kg/day	2.11	2.15	0.07	0.789
Feed to gain ratio, FCR	2.68^a^	2.55^b^	0.03	0.022

^*a*^ CON, control group; LP, *Lactobacillus*-fermented feed group. Different lowercase letters indicate statistically different between groups (*P* < 0.05).

### *Lactobacillus*-fermented feed decreased fecal valeric acid concentration

**Fig 1:**
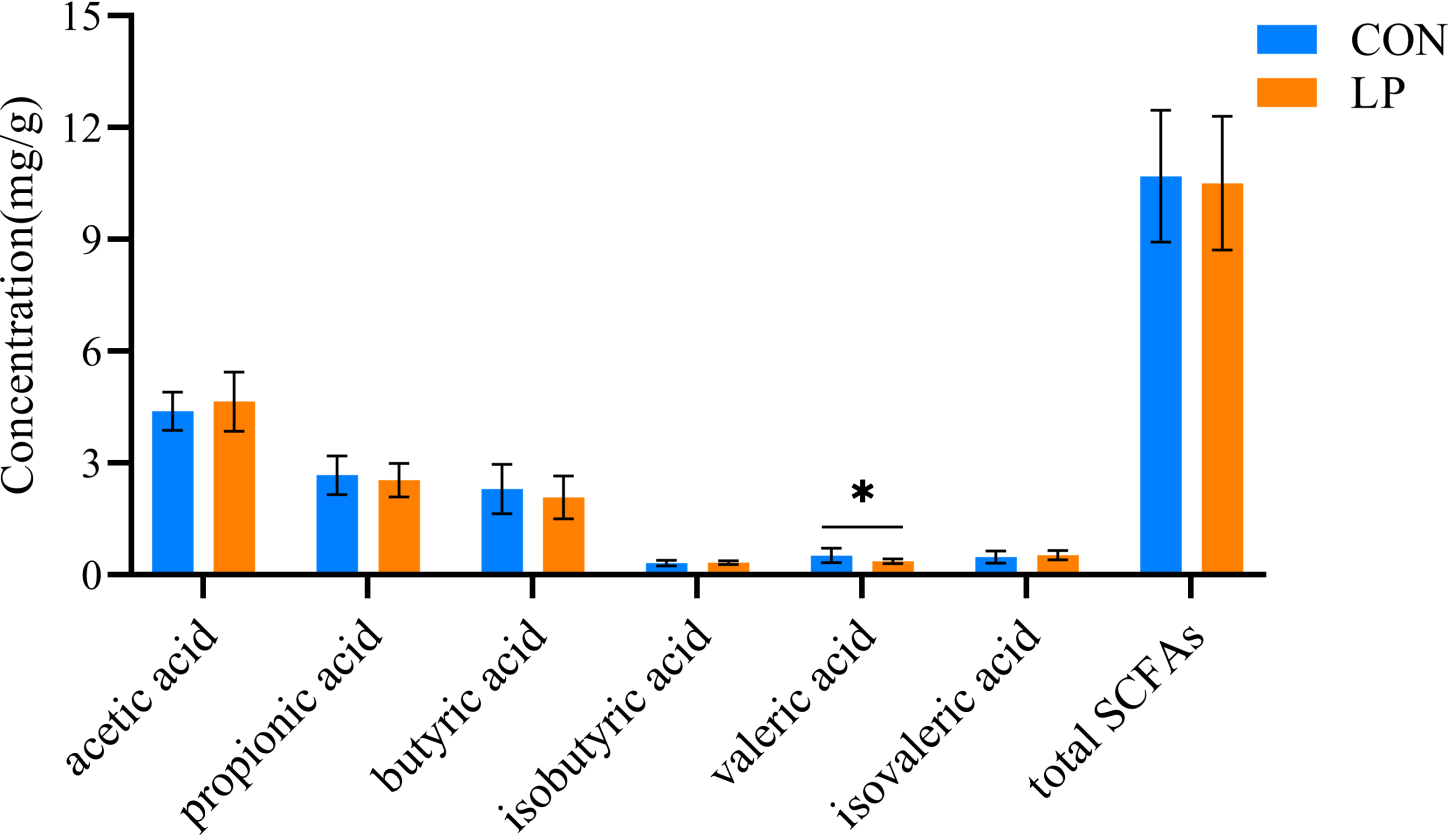
Effects of *Lactobacillus*-fermented feed on fecal compositions of short-chain fatty acids. CON, control group. LP, *Lactobacillus*-fermented feed group. Data are presented as “mean ± SEM” (*n* = 10). *P < 0.05.

Fecal SCFA profiles are shown in Fig. 1. Levels of valeric acid were substantially reduced in the LP group relative to CON (*P* < 0.05). In contrast, the levels of acetic, propionic, butyric, isobutyric, and isovaleric acids, as well as the total SCFA content, were comparable between the two groups (*P* > 0.05).

### *Lactobacillus*-fermented feed modulated composition of fecal microbiota

**Fig 2:**
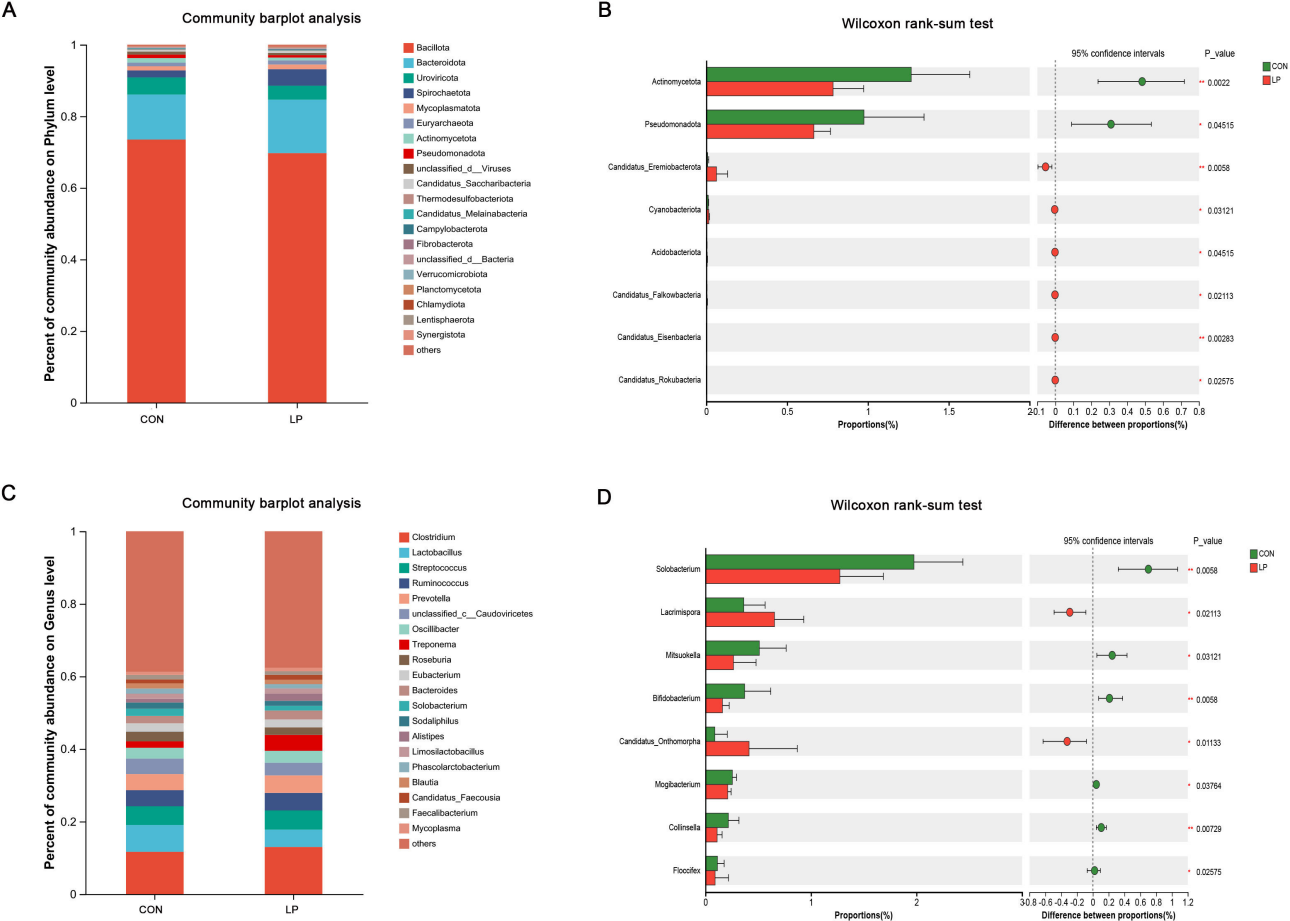
Effects of *Lactobacillus*-fermented feed on fecal microbial composition profiles. Relative abundance of phylum (**A**), genus (**C**). Difference of microbial composition at phylum (**B**) and genus (**D**) level. CON, control group. LP, *Lactobacillus*-fermented feed group. *P < 0.05, **P < 0.01.

The composition of fecal microbiota, including Bacteria, Archaea, Fungi, and DNA viruses, at the levels of both phylum and genus is illustrated in Fig. 2. At the phylum level (Fig. 2A), the dominant taxa in both CON and LP groups were Bacillota (formerly Firmicutes; 73.49% vs 69.69%), Bacteroidetes (12.56% vs 14.93%), Uroviricota (4.79% vs 3.90%), Spirochaetota (1.95% vs 4.59%), and Mycoplasmatota (1.26% vs 1.33%). Statistical analyses of phyla with relative abundance greater than 0.01% indicated that *Candidatus_Eremiobacterota* and Cyanobacteriota were substantially more abundant after LP treatment (*P* < 0.05), whereas Actinomycetota and Pseudomonadota were markedly reduced relative to CON (Fig. 2B).

At the genus level (Fig. 2C), the microbial communities in both groups were primarily composed of *Clostridium* (11.65% vs 12.98%), *Lactobacillus* (7.38% vs 4.78%), *Streptococcus* (5.15% vs 5.28%), *Ruminococcus* (4.43% vs 4.83%), *Prevotella* (4.43% vs 4.82%), *unclassified_c_Caudoviricetes* (4.28% vs 3.49%), and *Treponema* (1.77% vs 4.38%), with other genera each representing less than 4% of the total abundance. Further statistical evaluation of genera with relative abundance exceeding 0.1% revealed that *Lacrimispora* and *Candidatus_Onthomorpha* were substantially enriched following LP treatment (*P* < 0.05), whereas *Solobacterium* and *Mitsuokella* were markedly less abundant compared with CON (Fig. 2D).

### *Lactobacillus*-fermented feed modulated fecal metabolomics

**Fig 3:**
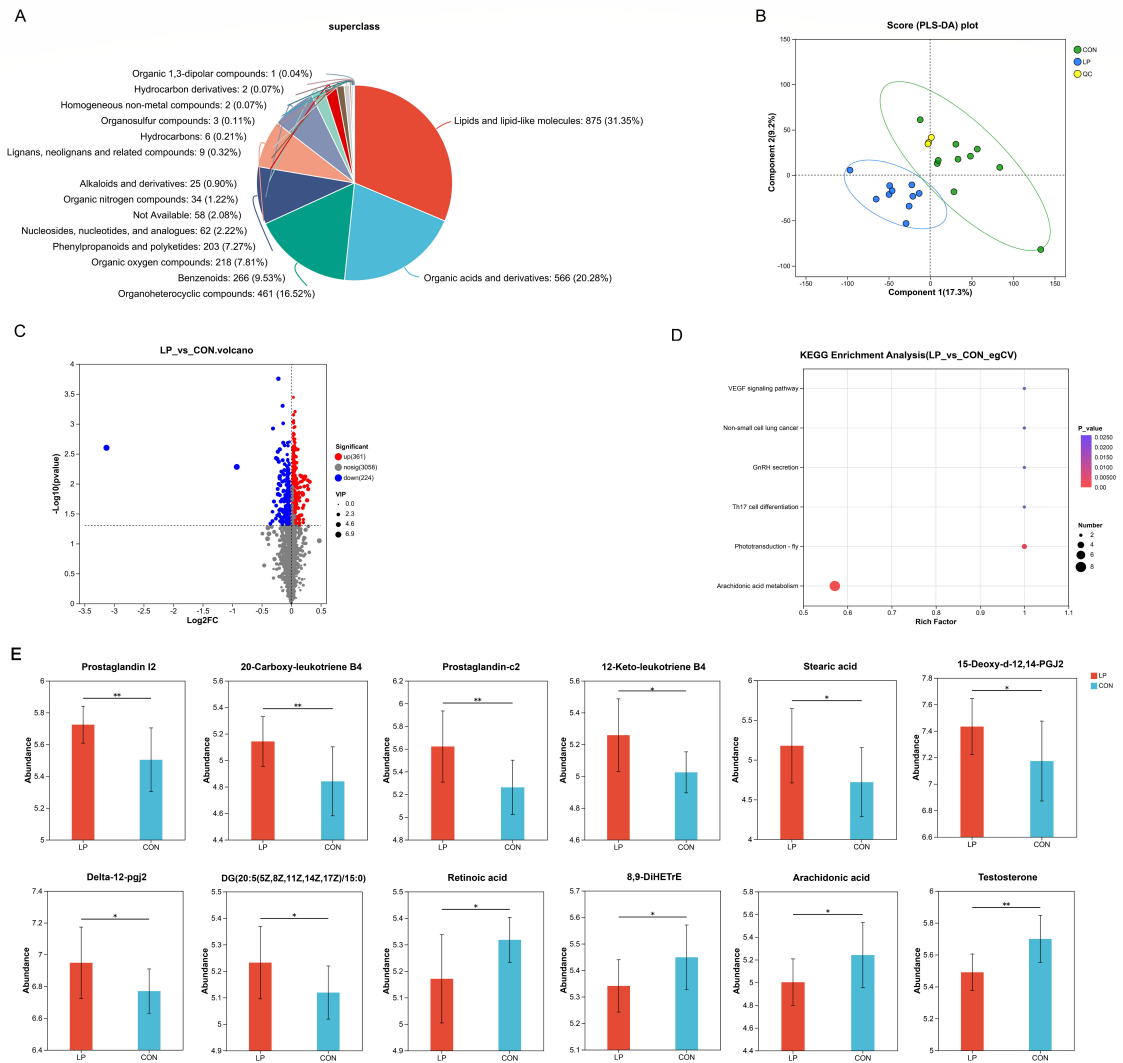
Effects of *Lactobacillus*-fermented feed on fecal metabolome of growing pigs. (**A**) Pie-super class. (**B**) Scatter plots of PLS-DA. (**C**) The volcano plot of differential metabolites. (**D**) The enriched KEGG in differential metabolites. (**E**) The box map of abundance of 12 differential metabolites between the CON and LP groups. CON, control group. LP, *Lactobacillus*-fermented feed group. *P < 0.05, **P < 0.01.

Overall, 3,643 metabolites were identified after LC-MS analysis. These consisted predominantly of lipids and lipid-like molecules (31.35%), organic acids and derivatives (20.28%), organoheterocyclic compounds (16.52%), and benzenoids (9.53%) at the superclass level (Fig. 3A). PLS-DA analysis demonstrated clear separation between the CON and LP groups under both positive and negative ion modes, indicating substantial differences in metabolite profiles (Fig. 3B). Using the thresholds of VIP > 1 and *P* < 0.05, 224 metabolites exhibited marked downregulation, while 361 showed substantial upregulation in the LP group relative to CON (Fig. 3C). KEGG analysis indicated marked enrichment of 12 differential metabolites in six pathways (Fig. 3D). Among these, arachidonic acid metabolism, phototransduction—fly, and Th17 cell differentiation were the most prominently altered pathways. Compared with the CON group, prostaglandin I2, 20-Carboxy-leukotriene B4, prostaglandin C2, 12-Keto-leukotriene B4, stearic acid, 15-Deoxy-d-12,14-PGJ2, delta-12-pgj2, and DG(20:5(5Z,8Z,11Z,14Z,17Z)/15:0, were substantially increased, whereas retinoic acid, 8,9-DiHETrE, arachidonic acid, and testosterone were markedly decreased following LP treatment (*P* < 0.05) (Fig. 3E).

### Microbiota and metabolites associated with growth performance and SCFAs

**Fig 4:**
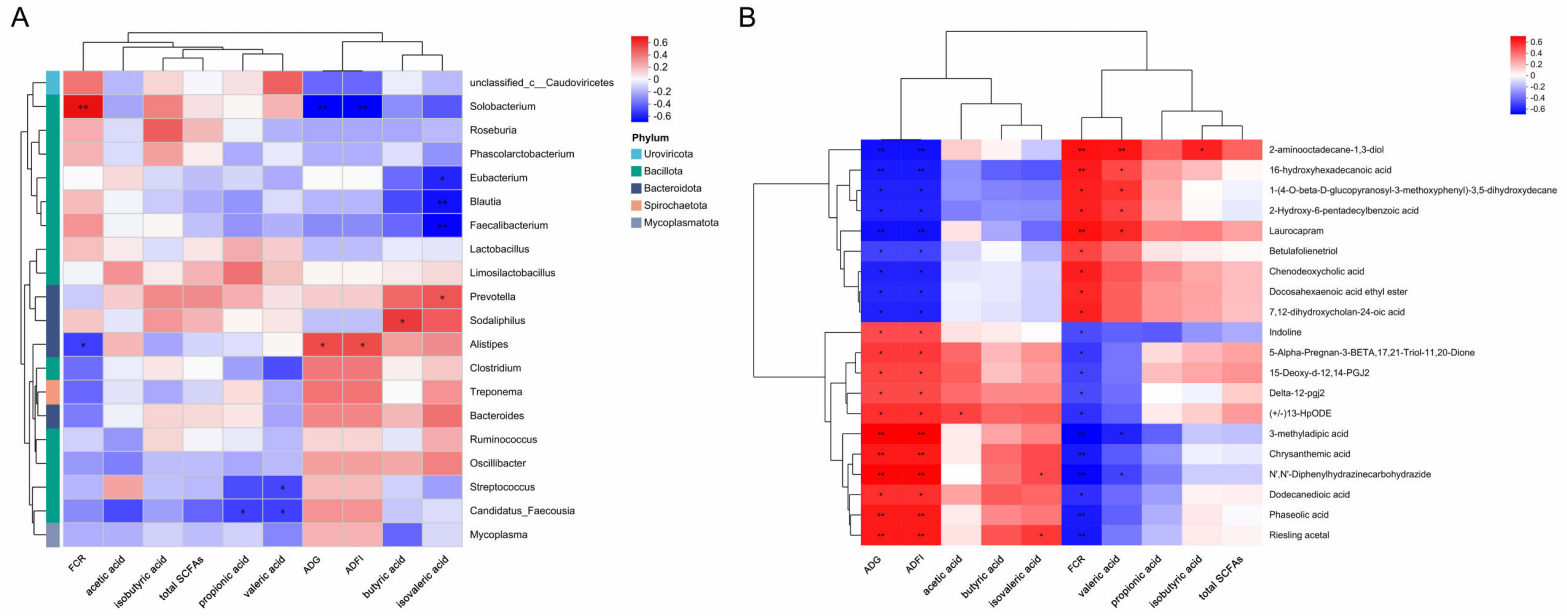
The correlation heatmap between fecal microbiota, fecal metabolites, and fecal SCFAs. (**A**) Spearman correlation between microbiota and VFAs. (**B**) Spearman correlation between metabolites and VFAs. *P < 0.05, **P < 0.01.

To further elucidate the associations among fecal microbiota, metabolite profiles, growth performance, and SCFA production in pigs receiving fermented feed, Spearman’s correlation analyses were conducted. As shown in Fig. 4A, FCR exhibited a positive association with *Solobacterium* abundance, whereas it was negatively associated with *Alistipes*. In contrast, both ADG and ADFI were positively linked with *Alistipes* abundance and negatively linked with *Solobacterium*. With respect to SCFAs, propionic acid concentrations were negatively associated with *Candidatus_Faecousia*, while valeric acid exhibited negative associations with *Streptococcus* and *Candidatus_Faecousia*. Butyric acid levels were positively correlated with *Sodaliphilus*. In addition, isovaleric acid concentration was positively associated with *Prevotella* abundance, but negatively with *Faecalibacterium*, *Blautia*, and *Eubacterium* levels.

Correlation analysis between differential metabolites and phenotypic traits (Fig. 4B) revealed that FCR was positively associated with downregulated metabolites, including the sphingoid base 2-aminooctadecane-1,3-diol, the hydroxylated fatty acid 16-hydroxyhexadecanoic acid, and the synthetic permeation enhancer laurocapram. Conversely, FCR was negatively associated with upregulated metabolites, such as the sesquiterpene riesling acetal, the plant stress metabolite phaseolic acid, the synthetic compound N′,N′-diphenylhydrazinecarbohydrazide, the monoterpenoid chrysanthemic acid, and the dicarboxylic acid 3-methyladipic acid. By contrast, ADG and ADFI exhibited the opposite correlation pattern relative to FCR. Furthermore, valeric acid concentration tended to be positively associated with downregulated metabolites and negatively with upregulated metabolites. Acetic acid concentration showed a positive association with the upregulated metabolite (±)13-HpODE. Similarly, isovaleric acid was positively associated with upregulated metabolites such as N′,N′-diphenylhydrazinecarbohydrazide and riesling acetal, whereas isobutyric acid was positively linked with the downregulated metabolite 2-aminooctadecane-1,3-diol.

## DISCUSSION

A substantial body of evidence indicates that dietary inclusion of fermented feed can enhance growth performance in swine (11, 12). Here, the inclusion of *Lactobacillus*-fermented feed markedly reduced FCR, suggesting improved feed efficiency. Although increases in ADG and ADFI were observed, these changes were not statistically significant, implying that the primary benefit was enhanced feed utilization rather than increased intake or growth rate. These findings support those of Hao et al. (13), who found a marked reduction in FCR in finishing pigs fed a diet containing 10% fermented mixed feed. The improvement in FCR may be attributed to enhanced nutrient utilization efficiency. *Lactobacillus*-fermented feed contains viable lactic acid bacteria along with fermentation-derived metabolites such as organic acids, enzymes, and extracellular polysaccharides. These components can improve feed palatability and nutrient availability, promote intestinal health, and facilitate digestion and absorption, ultimately contributing to improved production performance (14–16). Nevertheless, not all studies report consistent outcomes. For example, Xin et al. (17) observed no significant change in FCR despite improvements in ADG and ADFI in weaned pigs fed fermented liquid diets. Such discrepancies among studies may arise from differences in microbial strains, fermentation conditions, feeding strategies, animal genotype and developmental stage, dietary composition, and environmental factors (18, 19). The present study evaluated the effects of *L. curvatus* SQ13-fermented feed at a fixed inclusion dose. However, given that different strains or doses might result in different effects on growth performance, further research is required to explore these variables in greater depth.

SCFAs, including acetate, propionate, butyrate, isobutyrate, and valerate, are microbial metabolites produced through fermentation of dietary carbohydrates in the hindgut. These compounds can provide energy substrates to intestinal epithelial cells and are important in maintaining mucosal immunity, preserving epithelial barrier integrity, lowering intestinal pH to inhibit pathogenic microbes, and regulating host energy metabolism and inflammatory responses (20, 21). SCFA production is influenced by multiple factors, including dietary fiber composition, microbial community structure, and the presence of specific fermentative taxa (22). In this study, valeric acid concentration was reduced in pigs receiving the fermented feed, whereas the levels of other SCFA remained unchanged. Although valerate is typically associated with fiber degradation (23, 24), the selective reduction observed here suggests a specific metabolic shift rather than a general decline in fermentation activity. This may result from changes in microbial cross-feeding pathways (25) or reduced hindgut substrate availability due to improved feed efficiency. Despite the reduction in valerate, which has immune-modulating properties (26), the overall stability of SCFA indicates that the fermented feed supports normal intestinal function. Nonetheless, the precise metabolic pathways and microbial interactions responsible for these effects remain to be fully elucidated and warrant further investigation.

The gut microbiota is integral to host health, contributing to nutrient digestion and metabolism, protection against pathogenic invasion, and maintenance of the intestinal mucosal barrier (27, 28). Increasing evidence suggests that dietary supplementation with *Lactobacillus*-fermented feed can reshape the composition of the intestinal microbial community in pigs, thereby enhancing nutrient utilization and overall physiological status (3, 29). In the present study, notable shifts in microbial composition were observed following supplementation with fermented feed. At the phylum level, the relative abundances of *Candidatus_Eremiobacterota* and Cyanobacteriota were significantly elevated. Although these phyla are known to contain phototrophic representatives (30, 31), their increased abundance more likely reflects an indirect response of the gut microbiota to the fermented feed, driven by shifts in the intestinal environment (e.g., pH, metabolites, or nutrient availability), rather than a direct functional contribution from these phyla. Conversely, the abundance of Actinomycetota and Pseudomonadota was markedly reduced. Members of Actinomycetota are known to participate in lignocellulose degradation (32), whereas Proteobacteria (i.e., Pseudomonadota) are frequently considered indicators of gut dysbiosis, with elevated levels commonly associated with inflammation and metabolic disorders (33). These findings suggest that *Lactobacillus*-fermented feed may beneficially modulate gut microbial structure, potentially lowering the risk of intestinal inflammation and microbial imbalance. In terms of genera, the abundance of *Lacrimispora* and *Candidatus_Onthomorpha* rose markedly in pigs receiving fermented feed, whereas *Solobacterium* and *Mitsuokella* were markedly reduced relative to the control group. *Lacrimispora* is capable of fermenting carbohydrates into products, such as xylanase, ethanol, acetate, lactate, and butanol (34), and its enrichment may reflect enhanced digestive capacity. In contrast, *Solobacterium* has been associated with the upregulation of pro-inflammatory factors, potentially exacerbating inflammatory responses (35). Its reduced abundance in the present study may therefore contribute to improved intestinal health. Notably, *Solobacterium* abundance was positively linked to FCR and negatively correlated with ADG and ADFI, suggesting that proliferation of this genus may impair gut homeostasis and reduce feed efficiency. *Mitsuokella*, a member of the phylum *Bacillota*, is known to utilize lactic and acetic acids to produce butyric acid (36). The observed decrease in *Mitsuokella* abundance may be associated with the overall SCFA profile detected in this study, although most SCFAs showed no substantial differences between treatments, with the exception of valeric acid. Additionally, valeric acid levels were negatively correlated with *Streptococcus* and *Candidatus_Faecousia*. While previous work has demonstrated that *Lactobacillus*-fermented feed can enhance SCFA production and promote functional restructuring of gut microbial communities (10, 37), the precise regulatory mechanisms underlying these changes remain poorly understood and need to be analyzed at length.

The gut microbiota also produces a wide range of metabolites that are essential for host physiological functions and metabolic regulation (38). In this study, metabolomic profiling revealed substantial differences between control and fermented feed groups, with the majority of altered metabolites belonging to lipids and lipid-like compounds, organic acids and their derivatives, and organoheterocyclic molecules. KEGG analysis indicated that arachidonic acid metabolism, phototransduction—fly, and Th17 cell differentiation were the most prominently affected pathways in pigs receiving *Lactobacillus*-fermented feed. These alterations are likely driven by shifts in microbial composition, which in turn influence host metabolic processes (39). Arachidonic acid metabolism is central in regulating inflammation, immune responses, and vascular function (40, 41). It has been found that lactic acid bacteria can attenuate inflammatory responses through modulation of this pathway (42, 43). In particular, metabolites such as 15-Deoxy-d-12,14-PGJ2 and Δ12-PGJ2, which are derived from arachidonic acid, have well-documented anti-inflammatory properties (44, 45). In the current study, these metabolites were upregulated and showed negative correlations with FCR and positive correlations with ADG and ADFI, suggesting that *Lactobacillus*-fermented feed may improve feed efficiency by modulating anti-inflammatory metabolic pathways. However, as these conclusions are based on correlational analyses and have not been directly verified, further validation is required to confirm the underlying mechanisms. Phototransduction refers to the conversion of light signals into electrical signals. The observed increases in DG(20:5(5Z,8Z,11Z,14Z,17Z)/15:0) and stearic acid, along with decreased arachidonic acid levels, suggest that the fermented feed may modulate host lipid metabolism. Further research is needed to determine whether these metabolic changes are functionally linked to the annotated phototransduction pathway in the gut. In addition, Th17 cells play a critical role in immune regulation and inflammatory processes (46). The enrichment of pathways related to Th17 cell differentiation indicates that *Lactobacillus*-fermented feed may exert immunomodulatory effects by influencing T-cell-mediated responses.

### Conclusion

In summary, dietary supplementation with 0.3% *Lactobacillus*-fermented feed significantly improved feed conversion efficiency and reduced valeric acid concentration in growing pigs. Moreover, this dietary intervention altered gut microbial composition and modulated key metabolic pathways. Correlation analyses further indicated that FCR was positively associated with *Solobacterium* and downregulated metabolites, whereas it was negatively associated with *Alistipes* and upregulated metabolites. Despite these findings, the mechanistic basis by which *Lactobacillus*-fermented feed enhances feed efficiency remains to be fully elucidated. Overall, this study provides both experimental evidence and a theoretical framework supporting the application of *Lactobacillus*-fermented feed in swine production, while highlighting the need for further mechanistic research.

## Data Availability

The microbiome data are available in the NCBI Sequence Read Archive under BioProject accession number PRJNA1436636.
